# Proceedings of the 2008 MidSouth Computational Biology and Bioinformatics Society (MCBIOS) Conference

**DOI:** 10.1186/1471-2105-9-S9-S1

**Published:** 2008-08-12

**Authors:** Jonathan D Wren, Dawn Wilkins, James C Fuscoe, Susan Bridges, Stephen Winters-Hilt, Yuriy Gusev

**Affiliations:** 1 Arthritis and Immunology Research Program, Oklahoma Medical Research Foundation; 825 N.E. 13th Street, Oklahoma City, OK 73104-5005, USA; 2 Computer & Information Science Department, The University of Mississippi, University, MS 38677, USA; 3 Center for Functional Genomics, Division of Systems Toxicology; National Center for Toxicological Research, U.S. Food and Drug Administration, Jefferson, AR 72079, USA; 4 Department of Computer Science and Engineering, Mississippi State University, Box 9637, Mississippi State, MS 39762, USA; 5 Department of Computer Science, University of New Orleans, and The Research Institute for Children, 200 Henry Clay Ave., New Orleans, LA 70118, USA; 6 Department of Surgery, Health Sciences Center, The University of Oklahoma, Oklahoma City, OK 73104, USA

## Introduction

MCBIOS 2008 was held February 23–24, 2008 in Oklahoma City, Oklahoma at the Cox Convention Center in Bricktown. It was the best attended in the series of MCBIOS conferences (140 registrants) with the most participation (68 posters submitted). Informative and engaging keynote talks were delivered by Dr. Bruce Roe and Dr. Edward Dougherty. The full agenda is online at .

Student poster award winners were: Vinay Ravindrakumar of University of Arkansas for Medical Sciences (1st place), Quan Shi of Little Rock Central High School (2nd) and Brian Roux of the University of New Orleans (UNO) (3rd), with honorary mentions going to Murat Eren of UNO and Prashanti Manda of Mississippi State University (MSU). Student talk winners were: Daniel Quest of the University of Nebraska Medical Center (1st place), Nan Wang of MSU (2nd), and William Sanders of MSU (3rd).

## Proceedings summary

This year, 19 out of 27 submitted papers were accepted for inclusion in the official conference proceedings (70%), similar to the number published from MCBIOS 2007 [1-26]. Each paper was peer-reviewed by at least two reviewers. Our goal in peer-review for the Proceedings is to be inclusive enough to accurately reflect the scope of scientific work presented at the conference yet rigorous enough such that only the highest quality work presented is selected for inclusion in the official proceedings. The general themes of this year's proceedings papers fall into five categories, discussed below.

## Systems biology

Biological systems can be modeled as complex systems with many interactions between the components. One goal of emerging systems biology is to analyze very large complex biological networks such as protein-protein interactions, metabolism, and regulation to identify functional modules and to assign the functions to certain components of the system. Mutlu Mete *et al*. [27] devised a new methodology called SCAN (Structural Clustering Algorithm for Networks) that can efficiently find clusters or functional modules in complex biological networks, as well as hubs and outliers. In addition, nodes can be classified into various roles based on their structures. Interpretations of functional groups found by SCAN showed superior performance over CNM, a well-known modularity-based clustering algorithm.

Analysis of microarray gene expression data is challenging and may lead to biased or incomplete biological interpretations. To gain a more holistic (i.e., systemic) picture, it is essential to integrate a careful statistical approach with biological knowledge from various sources into the analysis. Mikhail Dozmorov *et al*. [28] present an integrative approach to microarray analysis and demonstrate how the various steps in their process support each other and refine the current model of cell-matrix interaction. With their method, they were able to identify inflammation and G-protein signaling as processes affected by the extracellular matrix.

Metastases are responsible for the majority of cancer fatalities. The molecular mechanisms governing metastasis are poorly understood, hindering early diagnosis and treatment. Unlike most previous studies, a study by Andrey Ptitsyn *et al*. [29] proposes an approach that puts into focus gene interaction networks and molecular pathways rather than separate marker genes. This study indicates that regardless of the tissue of origin, all metastatic tumors share a number of common features related to changes in basic energy metabolism, cell adhesion/cytoskeleton remodeling, antigen presentation and cell cycle regulation.

Circadian rhythm is a crucial factor in orchestration of plant physiology, keeping it in synchrony with the daylight cycle. Previous studies reported approximately 16% of plant genes behaved in a circadian fashion, while studies in mammals suggested circadian baseline oscillation in nearly 100% of genes. Andrey Ptitsyn [30] presents a comprehensive analysis of periodicity in two independent *Arabidopsis thaliana *data sets. This study indicates a more pervasive role of gene expression oscillation in the molecular physiology of plants than previously believed. Application of advanced algorithms identified circadian baseline oscillation in almost all plant genes as well as a complex orchestration of gene expression timing in important biological pathways.

## OMICS

Chromatography coupled to mass spectrometry is a powerful way to resolve and compare the relative abundance of chemical compounds within heterogeneous biological samples. However the resulting data sets are 2 or 3-dimensional, presenting formidable obstacles to peak alignment – a process required to ensure sample comparison is conducted appropriately. The first dimension of separation is chromatographic elution time, which varies from run to run for each molecular species. To solve this problem, Minho Chae *et al*. [31] developed an iterative block-shifting approach that adjusts for variation in retention time without distorting peak area. They first matched chemically identical peaks based on both retention-time and mass-spectral information. Non-peak regions of each chromatogram were stretched or compressed to align peaks with a reference chromatogram, thus preserving the shapes of matched peaks. Their approach compared favorably to other approaches, and was superior in preservation of peak area.

Also, in the proceedings, Tianxiao Huan *et al*. describe Proteolens, a new tool to navigate and visualize biological networks [32].

## Microarray studies

Microarrays are a powerful technology and an area of active research interest in bioinformatics, with a focus on the development of novel methods for analysis and interpretation of experiments [33-49]. This year's proceedings reflect this area of active research interest with several reports that focus on the development of methods and analysis of microarray data.

Microarray-based molecular signatures have played an increasing role in diagnosis, prognosis and risk/safety assessments, the first step of which is to identify a set of informative genes. Zhenqiang Su *et al*. [50] investigate a new gene selection approach to identify informative genes. The rationale of the approach is that informative genes should consistently be significantly differentially expressed for different variations of sample size. Genes exhibiting significance throughout the iterations are considered a Very Important Pool (VIP) of genes. It was found that the genes identified by the VIP method, but not by the p-value ranking approach, are also related to the disease investigated, and these genes are part of the pathways derived from the common genes shared by both the VIP and p-ranking methods. Moreover, the binary classifiers built from these genes are statistically equivalent to those built from the top 50 p-value ranked genes in distinguishing different types of samples. Therefore, the VIP gene selection approach could identify additional subsets of informative genes that would not always be selected by the p-value ranking method.

The paper by Taewon Lee *et al*. [51] presents a method to test the significance of expression changes within a group of genes, while considering the correlation structure among genes in each group. This method enables the rapid detection of gene expression changes, indicating altered cell functions or pathways, and facilitates the interpretation of the data. Application of the method to real data shows that it is an improved, practical method to evaluate the effects of treatments on functional classes of genes, such as those based on Gene Ontology descriptors.

Also in the proceedings, Arun Rawat *et al*. report on a method of microarray graph mining to derive co-expressed genes [52], and Leming Shi *et al*. report on an impressively large study of the reproducibility of gene lists for microarray experiments, and conclude with recommendations for detecting significant differential expression [53].

## Genomic analysis

As more and more genomes become fully sequenced in the coming years, gene identification is still a limiting factor to scientific discovery. Since a significant proportion of genes exist as members of families of genes with related functions, Ronald Frank *et al*. [54] have employed a strategy to identify these gene family members using patterns indicating negative selection pressure on the coding region. The authors tested the strategy on several well-characterized gene families from Arabidopsis thaliana and report their success in correctly identifying several members of each gene family starting with one known member and using only EST data.

Highly accurate and reproducible genotype calling are paramount for genome-wide association studies (GWAS), since errors introduced by calling algorithms can lead to inflation of false associations between genotype and phenotype. Most genotype calling algorithms currently used for GWAS are based on multiple arrays, consisting of many samples. Huixiao Hong *et al*. [55] observed that batch size and composition affect the genotype calling results in GWAS using the algorithm BRLMM. The larger the differences in batch sizes, the larger the effect. The more homogenous the samples in the batches, the more consistent the genotype calls. The inconsistency propagates to the lists of significantly associated single nucleotide polymorphisms identified in downstream association analysis. Thus, uniform and large batch sizes should be used to make genotype calls for GWAS. In addition, samples of high homogeneity should be placed into the same batch.

The cellular machinery by which genes are expressed is both complex and an active area of recent bioinformatics research [56-66]. A first step in understanding this process is to locate the binding positions of transcription factors over the chromosome. Since the search space is large, advanced computational tools play a central role in solving this problem. Despite the development of nearly two hundred tools to elucidate transcription factor binding sites, much controversy still remains on how to build methods with high sensitivity and specificity. Central in this debate is determining the factors that will improve the quality of computational predictions. The paper by Daniel Quest *et al*. [67], presents a novel benchmarking strategy to automate and evaluate methods designed to detect transcription factor binding sites. The strategy allows researchers, for the first time, to evaluate transcription factor detection methods on the genome scale. In particular, researchers can vary the data, algorithms, parameters and transcription factor binding site representations to determine the method best suited to their problem of interest. The proposed platform allows for rapid evaluation of deficits in current models and paves the way to develop new tools to overcome these problems.

Also, the Garner Lab extends their work on predicting the impact of single nucleotide polymorphisms (SNPs) in a paper by Vinayak Kulkarni *et al*. [68], and Jerzy Zielinski *et al*. report on a method of analyzing genomic sequences by a time-dependent autoregressive moving average [69].

## Miscellaneous

Text-mining is an area of bioinformatics whereby identification and analysis of trends in text is done computationally [70-78]. To this end, Cory Giles and Jonathan Wren developed a method of identifying directional relationships within text (e.g., chemical X increases heart rate, or gene Y elevates inflammation) using natural language processing (NLP) [79]. Their goals were also to make their system scalable to large bodies of text (e.g. MEDLINE has 18 million records and counting), as well as understanding how much apparent contradiction takes place when attempting to extract isolated facts from within a greater context from these huge bodies of text.

Christopher Bottoms and Dong Xu study atom-naming conventions in the Protein Data Bank and find that some names are assigned ad hoc, resulting in duplicate names and creating problems for standardization and data-mining [80].

In [81], Roux and Winters-Hilt describe Hybrid SVM/HMM structural sensors for use in analysis of stochastic sequential data. They begin with a novel approach to classification using Support Vector Machines and Markov Models with application to detecting Intron-Exon and Exon-Intron (5' and 3') splice sites. The approach also includes the application of Shannon Entropy based analysis of the stochastic datasets to detect minimal data components for feature extraction. Results are presented for a variety of eukaryotic species.

In the Winters-Hilt group, work continues on developing nanopore detector signal analysis via machine learning methods for classification and knowledge discovery. In [82], Churbanov and Winters-Hilt describe the application of a distributed Mixture of Hidden Markov Models (MHMMs) to the problem of channel current blockade clustering and associated analyte classification. The distributed MHMM provides a feature extraction that is equivalent to that of the sequential HMM with a speedup factor approximately equal to the number of independent CPUs operating on the data.

## Future meetings

The Sixth annual MCBIOS Conference will be held in Starkville, Mississippi in early spring, 2009. See  for further information on MCBIOS and future meetings. MCBIOS and OKBIOS are both regional affiliates of the International Society for Computational Biology .

## Competing interests

The authors declare that they have no competing interests.

## Authors' contributions

All authors served as co-editors for these proceedings, with JDW serving as Senior Editor. All authors helped write this editorial. The findings and conclusions in this report are those of the authors and do not necessarily represent the views of the Food and Drug Administration.
